# Mechanism of leptin-NPY on the onset of puberty in male offspring rats after androgen intervention during pregnancy

**DOI:** 10.3389/fendo.2023.1090552

**Published:** 2023-03-28

**Authors:** Jingqi Liu, Yongting Yuan, Xingwang Peng, Yuanyuan Wang, Ruiyao Cao, Yun Zhang, Lianguo Fu

**Affiliations:** Department of Child and Adolescent Health, School of Public Health, Bengbu Medical College, Bengbu, China

**Keywords:** leptin, NPY, puberty onset, androgen, GnRH

## Abstract

**Objectives:**

The time of onset of puberty has been increasingly earlier, but its mechanism is still unclear. This study aimed to reveal the mechanism of leptin and NPY in the onset of puberty in male offspring rats after androgen intervention during pregnancy.

**Methods:**

Eight-week-old specific pathogen-free (SPF) healthy male Sprague−Dawley (SD) rats and 16 female SD rats were selected and caged at 1:2. The pregnant rats were randomly divided into the olive oil control group (OOG) and testosterone intervention group (TG), with 8 rats in each group. Olive oil and testosterone were injected from the 15th day of pregnancy, for a total of 4 injections (15th, 17th, 19th, 21st day). After the onset of puberty, the male offspring rats were anesthetized with 2% pentobarbital sodium to collect blood by ventral aorta puncture and decapitated to peel off the hypothalamus and abdominal fat. Serum testosterone (T), free testosterone (FT), dihydrotestosterone (DHT), dehydroepiandrosterone (DHEA), sex hormone binding globulin (SHBG), and leptin were detected by ELISA, and then the free androgen index (FAI) was calculated. The mRNA levels of androgen receptor (AR), estrogen receptor α (ERα), NPY, leptinR, and NPY2R in the hypothalamus and abdominal fat were detected by RT−PCR. Protein expression levels of AR, ERα, NPY, leptinR, and NPY2R in the arcuate nucleus (ARC) of the hypothalamus were detected by immunohistochemistry.

**Results:**

The time of onset of puberty was significantly earlier in the TG than in the OOG (*P<* 0.05) and was positively correlated with body weight, body length, abdominal fat, and leptinR mRNA levels in adipose tissue in the OOG (*P<* 0.05), while it was positively correlated with serum DHT and DHEA concentrations and FAI and AR mRNA levels in the hypothalamus in the TG (*P<* 0.05). The NPY2R mRNA level and protein expression levels of ERα, NPY2R, and leptinR in the TG were significantly higher than those in the OOG, while the protein expression levels of AR and NPY in the TG were significantly lower than those in the OOG (*P<* 0.05).

**Conclusions:**

Testosterone intervention during pregnancy led to an earlier onset of puberty in male offspring rats, which may render the male offspring rats more sensitive to androgens, leptin, and NPY at the onset of puberty.

## 1 Introduction

Puberty is defined as the “first reproductive period”, which marks the maturity of the genitals, the development of secondary sexual characteristics, the acceleration of linear growth, emotional changes, and the occurrence of first spermatorrhea or menarche (1). The onset time of puberty has shown an increasing trend of being earlier worldwide (2). In the past 150 years, the average age of menarche of European children has declined from approximately 16–17 years in the middle of the 19th century to 12 years in the middle of the 20th century (3, 4), which is estimated to be advanced by 3 years every 100 years (5). A cohort study of Swedish boys showed that the starting age of puberty was 1.5 months earlier for every decade increase in birth year (6). Moreover, the average age at which Danish boys initiated puberty was 3 months earlier from 2006 to 2008 than before (7), and that of Chinese urban boys was 2 years earlier from 2003 to 2005 than 1979 (8). The early onset of puberty not only affects mental health in children and adolescents but also increases the risk of certain diseases in adulthood, such as metabolic syndrome, cardiovascular disease, osteoporosis, and testicular cancer (9, 10), which has developed into a serious public health problem.

With the development of ‘Fetal Origin of Adult Disease’ (FOAD) and ‘Development Origin of Health and Disease’ (DOHaD) (11, 12), an increasing number of studies have linked the endocrine environment during pregnancy to the long-term effects on future generations (13–16). With lifestyle changes and the effects of exogenous endocrine disruptors (17), the proportion of women with high androgen levels during pregnancy was significantly increased (18–20). Exposure to prenatal high androgen environments may lead to changes in hormone levels and pubertal development in offspring (21).

Leptin is a protein product of the obesity (Ob) gene and plays an important role in pubertal development and reproduction (22). Recent studies have found that leptin can regulate the synthesis and release of GnRH in the hypothalamic arcuate (ARC) nucleus (23). GnRH secretion is insufficient in mice lacking the leptin gene (24). Studies have directly shown that leptin can trigger the beginning of puberty in boys (25). However, studies have found that there is no leptin receptor expression or very little expression in ARC nucleus GnRH neurons (26). Therefore, leptin cannot directly act on GnRH neurons, but it is involved in regulating puberty onset (27, 28). However, leptin may indirectly affect the activity of GnRH neurons by affecting NPY neurons (29). NPY was identified as the main regulator of GnRH pulse secretion (30), which is the ‘gatekeeper’ of adolescence (31). As a regulator, NPY neurons are located in the ARC (32), which can directly exert an inhibitory effect on GnRH neurons (33). This effect is achieved by the direct binding of NPY to NPY1R on GnRH neurons (34). Moreover, recent studies have demonstrated that leptin receptors exist in NPY neurons (24), which indicates that leptin can directly regulate NPY neurons (35) and thus play an important role in regulating reproduction (36).

However, whether high androgen exposure during pregnancy can participate in puberty onset by affecting the levels of leptin and NPY in offspring has rarely been reported. Therefore, this study administered androgen intervention to maternal rats during pregnancy to shed light on the mechanism of leptin and NPY in puberty onset of offspring male rats after androgen intervention during pregnancy, which provides a powerful clue for exploring the relationship between prenatal androgen and the onset of puberty in male offspring.

## 2 Participants and methods

### 2.1 Rats, diet, and experimental procedures

All animal care and experimental procedures were approved by the Ethics Committee of Bengbu Medical College ([2018] No.032). A total of 16 eight-week-old healthy SD female rats (200–250 g) and 8 male rats (300–330 g) were purchased from Jinan Peng Yue Experimental Animal Reproduction Co., Ltd. Animals were raised in a clean animal room with relative temperature (25 ± 2°C), relative humidity (40–70%), free light, and free access to water and diet. After a week of adaptive feeding, the female and male rats were caged at 9 o’clock every night at a ratio of 1:1, and vaginal plugs were observed at 9 o’clock the next morning to determine whether the rats were pregnant. Then, the pregnant female rats were randomly divided into the olive oil control group (OOG) and testosterone intervention group (TG), with 8 rats in each group. The pregnant female rats were treated with subcutaneous injection on the back of the neck starting from the 15th day of pregnancy, for a total of 4 injections (15th, 17th, 19th, 21st day). TG was given 2 mL of 2.5 g/ml testosterone solution (dissolved in olive oil), and OOG was given the same dose of olive oil. After the birth of the offspring, 36 male offspring rats were taken, which were raised to 21 days (PND21) and then weaned. From PND21, the body weight of the rats was recorded daily, and the genitals were observed. The foreskin separation of the male rats was used as a sign of the onset of puberty. The number of days taken to initiate puberty was recorded in all experimental rats. Twelve rats (TG : OOG = 1:1) were used for the detection of body shape indicators, serum hormone levels, and the mRNA of hypothalamic neuroendocrine function genes. Then, 6 rats (TG : OOG = 1:1) were used to detect the expression of hypothalamic neuroendocrine functional proteins by immunohistochemistry.

### 2.2 Sample collection

After anesthesia with 2% pentobarbital sodium solution at a dose of 3 ml/kg, we punctured the abdominal aortas of the rats to collect blood, and the blood was centrifuged to obtain the upper serum, which was then stored at -80 °C. We decapitated the rats, stripped their hypothalamus and abdominal fat, immediately placed them in liquid nitrogen and transferred them to the refrigerator at -80 °C at the end of the experiment. The above samples were all operated on ice.

### 2.3 Body shape indices and wet weight of abdominal fat in rats

When signs of puberty onset were observed, the rats’ body weight, body length, anal-genital distance (AGD), abdominal fat, and abdominal fat coefficient were measured. The body weight was measured with an electronic scale and was accurate to 0.1 g; body length refers to the distance from the tip of the nose to the superior margin of the anus, which was measured accurately to 0.1 cm; the AGD was measured by a Vernier caliper after anesthesia in rats and was accurate to 0.01 mm. Finally, abdominal fat was measured using an analytical balance and was accurate to 0.01 g; abdominal fat coefficient (%) = [abdominal fat weight (g)/body weight (g)] × 100%.

### 2.4 Detection of serum biochemical indices

The concentrations of T, FT, DHT, DHEA, SHBG, and leptin in the male rat serum were determined using ELISA with rat T (cat. no. CSB-E05100r, CUSABIO, Wuhan, China), rat FT (cat. no. CSB-E05097r, CUSABIO), rat DHT (cat. no. CSB-E07879r, CUSABIO), rat DHEA (cat. no. CSB-E08227r, CUSABIO), rat SHBG (cat. no. CSB-E12118r, CUSABIO) and rat leptin ELISA kits (cat. no. CSB-E07433r, CUSABIO). FAI = [T (ng/ml)/SHBG (ng/ml)] × 100.

### 2.5 Isolation of RNA and real-time PCR

Total RNA was extracted from the hypothalamus and abdominal adipose tissue by the TRIzol method (Thermo Scientific, Shanghai, China). For the detection of RNA concentration, we used 1 μg total RNA reverse transcription cDNA according to the instructions of the kit manufacturer (Thermo Scientific), and the mRNA levels of AR, ERα, NPY, leptinR, and NPY2R in the above tissues were detected by quantitative real-time PCR. The gene-specific primer sequences were designed by Shanghai Generay Biotech Co., Ltd., and all the primer sequences are shown in **Table 1**. The polymerase chain reaction mixture was 15 μl, and the reaction system contained 7.5 μl of 2×qPCR Mix, 1.5 μl of 2.5 μM gene primer, 2.0 μl of reverse transcription product, and 4.0 μl of ddH2O. The three-step PCR amplification protocol was as follows: predenaturation, 95°C 10 min; cycle, 95°C 15 s → 60°C 60 s, a total of 40 cycles; melting curve, 60°C → 95°C, heating 0.3°C every 15 s.

**Table 1:** PCR primer sequences.

Primer name (-S upstream primer; -A downstream primer)	Primer sequence (5’-3’)
AR-S	TGGGACCTTGGATGGAGAACT
AR-A	GGCACATAGATACTTCTGTTTCCC
ERα-S	GTTTGCTCCTAACTTGCTCTTGG
ERα-A	TCAAGGTGCTGGATAGAAATGTG
NPY-S	CTCTGCGACACTACATCAATCTCA
NPY-A	CCTTGTTCTGGGGGCATTT
Leptin Receptor-S	GGAAACACAGGGGTCCA
Leptin Receptor-A	TAGCAGCATCAACACCGA
NPY Receptor-S	ACAGTGAACTTTCTCATAGGCAACC
NPY Receptor-A	CAGAACTGACACACATTGAAGGAAC

### 2.6 Immunohistochemical analysis

Hypothalamic tissue was fixed with 10% formalin and embedded in paraffin. The hypothalamus paraffin blocks were cut into 4 μm thick sections and placed in a repair box filled with citric acid antigen repair buffer (PH 6.0) for antigen retrieval in a microwave oven. After natural cooling, the slides were placed in PBS (PH 7.4), washed on a decolorized shaker 3 times for 5 minutes each time and then incubated in 3% hydrogen peroxide for 10 minutes to block endogenous peroxidase. Subsequently, 3% BSA was added to cover the tissue uniformly and sealed at room temperature for 30 min. The blocking solution was removed, and PBS was dropped in a certain proportion of the primary antibody (rabbit polyclonal antibody, Servicebio, Wuhan, China) on the slice. The samples were then placed in a wet box and incubated overnight at 4°C. Then, the cells were incubated with a biotinylated secondary antibody (rabbit polyclonal antibody, Servicebio, Wuhan, China) and placed at room temperature for 50 min. A DBA kit was used to observe the immune response at room temperature for 2 minutes. Finally, hematoxylin staining was used for microscopic examination. Integrated optical density (IOD) was used to describe the relative expression of AR, ERα, leptinR, NPY, and NPY2R proteins. The IOD was repeated three times for each sample using Image-Pro Plus software, and the average value was taken.

### 2.7 Statistical analysis

The statistical analysis was performed in SPSS 23.0 software. First, because of the inconsistency of the sampling time of onset of puberty, we standardized the data according to the sampling time [standardized index = (index before standardization/time of onset of puberty) × average time of onset of puberty] and carried out logarithmic conversion of the data. Measurement data are described as the mean ± standard deviation. The differences in morphological development indices, sex hormones, and mRNA between TG and OOG were analyzed by independent-sample *t test*, while the differences in immunohistochemical indices between TG and OOG were analyzed by the mixed linear model. Pearson correlation analysis was used to analyze the correlation. *P<* 0.05 was considered statistically significant.

## 3 Results

### 3.1 Effect of testosterone intervention during pregnancy on the time of onset of puberty in offspring male rats

**Figure 1:**
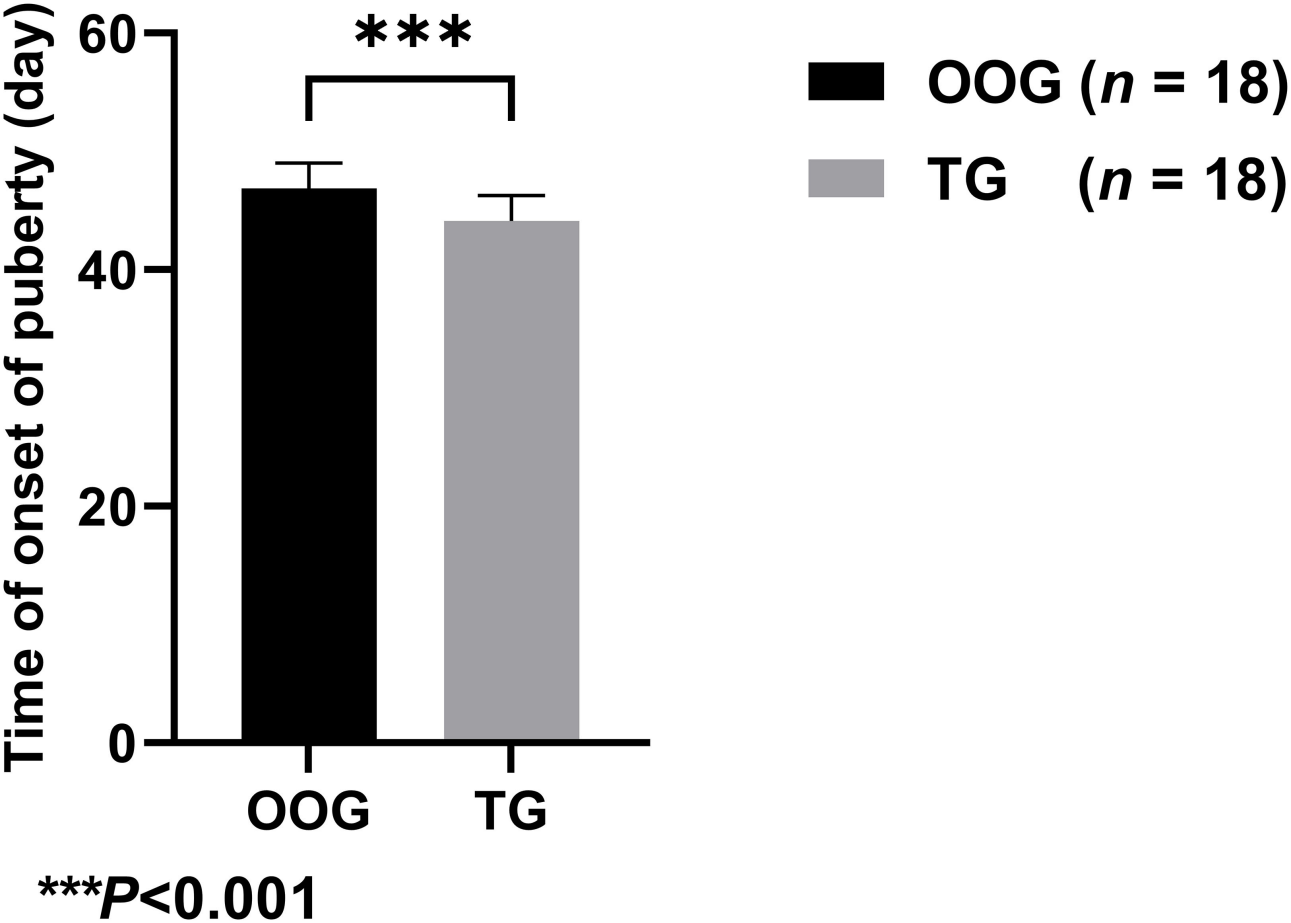
Comparison of the time of onset of puberty between OOG and TG.

**Figure 1** shows that the time of onset of puberty of male offspring in the TG (44.11 ± 2.14 days; *n* = 18) was significantly lower than that in the OOG (46.89 ± 2.14 days; *n* = 18), which suggested that testosterone intervention during pregnancy may lead to earlier time of onset of puberty of male offspring (*P*< 0.05).

### 3.2 Effects of testosterone intervention during pregnancy on morphological development and blood circulating sex hormone levels in offspring male rats

**Table 2** shows that there were no significant differences in body weight, body length, abdominal fat, abdominal fat coefficient, T, FT, DHT, DHEA, SHBG, FAI, or leptin between TG and OOG (*P* > 0.05). The results of correlation analysis showed that the time of onset of puberty in the OOG was positively correlated with body weight, body length, and abdominal fat (*P<* 0.05), while in the TG, the time of onset of puberty was positively correlated with serum DHT and DHEA concentrations and FAI (*P<* 0.05) and negatively correlated with SHBG concentration (*P<* 0.05). See **Table 3** for details.

**Table 2:** Comparison of body shape indices and blood circulating sex hormone indices in male offspring between OOG and TG ( $\bar{x}$ ± *s*).

Indices	OOG (*n* = 6)	TG (*n* = 6)	*t*	*P*
S Body weight (g)	194.762±15.998	191.768±38.97	0.17	0.87
S Body length (cm)	18.775±0.459	18.768±1.951	0.01	0.99
S Abdominal fat (g)	1.388±0.492	1.023±0.382	1.43	0.18
S Abdominal fat coefficient	0.700±0.208	0.520±0.138	1.77	0.11
Sln T (ng/ml)	1.303±0.854	0.651±0.503	1.61	0.14
Sln FT (pg/ml)	2.360±0.785	1.662±0.465	1.88	0.09
Sln DHT (pg/ml)	5.699±0.691	5.031±0.323	2.15	0.06
Sln DHEA (ng/ml)	0.158±0.762	-0.475±0.618	1.58	0.15
Sln SHBG (ng/ml)	6.592±0.250	6.679±0.578	-0.34	0.74
Sln Leptin (ng/ml)	-2.264±1.290	-2.979±0.264	1.33	0.21
Sln FAI	-0.716±0.820	-1.365±0.712	1.46	0.17

**Table 3:** Correlation between body shape indices, serum sex hormone levels and the time of onset of puberty in male offspring of OOG and TG.

Variables	Body weight	Body length	Abdominal fat	T	FT	DHT	DHEA	SHBG	FAI	Leptin
OOG										
Puberty onset time	0.821*	0.891*	0.844*	0.414	0.364	0.398	0.394	0.695	0.286	-0.299
Body weight	1	0.819*	0.985**	0.47	0.384	0.455	0.454	0.448	0.432	0.258
Body length		1	0.764	0.753	0.710	0.740	0.744	0.763	0.655	-0.148
Abdominal fat			1	0.353	0.264	0.338	0.333	0.389	0.308	0.204
T				1	0.987**	1.000**	0.998**	0.429	0.984**	0.077
FT					1	0.987**	0.981**	0.468	0.970**	0.067
DHT						1	0.998**	0.413	0.986**	0.078
DHEA							1	0.424	0.983**	0.066
SHBG								1	0.301	-0.347
FAI									1	0.230
TG										
Puberty onset time	-0.747	-0.718	-0.595	0.789	0.800	0.815*	0.812*	-0.887*	0.951**	0.029
Body weight	1	0.908*	0.830*	-0.277	-0.282	-0.252	-0.286	0.936**	-0.624	0.278
Body length		1	0.957**	-0.307	-0.305	-0.339	-0.262	0.790	-0.583	0.204
Abdominal fat			1	-0.162	-0.17	-0.203	-0.092	0.679	-0.427	0.007
T				1	0.998**	0.954**	0.981**	-0.467	0.919**	0.238
FT					1	0.953**	0.981**	-0.48	0.921**	0.278
DHT						1	0.955**	-0.465	0.877*	0.192
DHEA							1	-0.518	0.924**	0.155
SHBG								1	-0.776	0.188
FAI									1	0.067

### 3.3 Effects of testosterone intervention during pregnancy on mRNA levels of AR, ER-α, leptinR, NPY, NPY2R

Testosterone intervention during pregnancy resulted in markedly elevated mRNA levels of NPY2R in the hypothalamus compared with the OOG, whereas the mRNA levels of leptinR in the fat were significantly downregulated (*P*< 0.05; **Table 4**). There was a statistically positive correlation between the time of onset of puberty and leptinR mRNA levels in adipose tissue in the OOG (*R* = 0.840, *P<* 0.05) but not with other indicators (**Table 5**). Nevertheless, after testosterone intervention, there was only a statistically positive correlation between the time of onset of puberty and the level of AR mRNA in the hypothalamus (*R* = 0.883, *P*< 0.05; **Table 5**).

**Table 4:** Comparison of the content of functional gene mRNA in male offspring between OOG and TG ( $\bar{x}$ ± *s*).

Indices	OOG (*n* = 6)	TG (*n* = 6)	*t*	*P*
Hypothalamus				
S AR	1.006±0.609	0.809±0.415	0.653	0.529
S ER-α	1.015±0.997	0.485±0.436	1.195	0.272
S LeptinR	0.990±0.153	1.248±0.252	-2.149	0.057
S NPY	0.995±0.486	1.442±1.010	-0.978	0.351
S NPY2R	0.997±0.315	2.719±1.070	-3.779	<0.05
Fat				
S Leptin	0.997±0.498	0.671±0.372	1.283	0.228
S LeptinR	0.981±0.300	0.650±0.093	2.584	<0.05

**Table 5:** Correlation between hypothalamic functional gene mRNA levels and the time of onset of puberty in offspring of OOG and TG.

Variables	Hypothalamic AR	Hypothalamic ERα	Hypothalamic LeptinR	Hypothalamic NPY	Hypothalamic NPY2R	Fat Leptin	Fat LeptinR
OOG							
Puberty onset time	-0.411	-0.546	0.42	-0.092	-0.555	-0.209	0.840*
Hypothalamic AR	1	0.960**	-0.446	-0.103	0.72	-0.013	-0.168
Hypothalamic ERα		1	-0.365	0.137	0.609	0.085	-0.409
Hypothalamic LeptinR			1	0.568	-0.521	0.538	0.202
Hypothalamic NPY				1	-0.58	0.208	-0.505
Hypothalamic NPY2R					1	0.176	-0.065
Fat Leptin						1	-0.223
TG							
Puberty onset time	0.883*	-0.098	0.155	0.037	0.727	-0.256	0.377
Hypothalamic AR	1	-0.073	0.193	0.043	0.807	-0.278	0.044
Hypothalamic ERα		1	0.544	0.911*	-0.419	0.745	-0.361
Hypothalamic LeptinR			1	0.838*	0.245	0.388	0.282
Hypothalamic NPY				1	-0.164	0.652	-0.06
Hypothalamic NPY2R					1	-0.285	0.441
Fat Leptin						1	-0.100

### 3.4 Effects of testosterone intervention during pregnancy on the expression of AR, ERα, NPY, NPY2R, and leptinR

**Figure 2:**
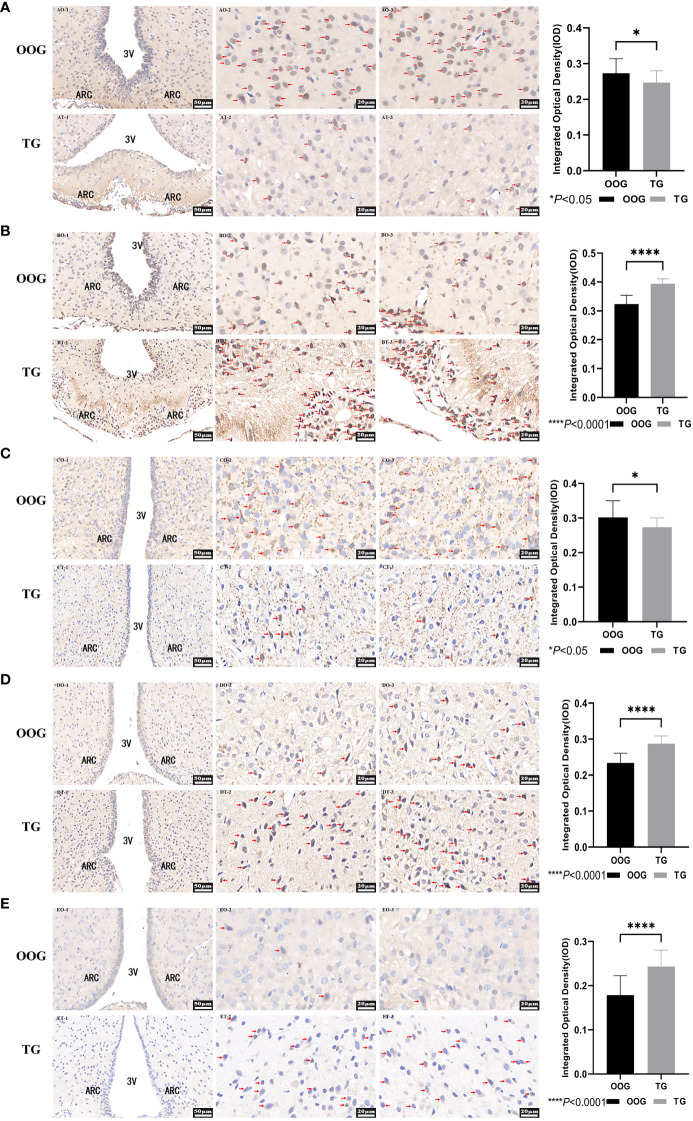
Protein expression of AR, ERα, NPY, NPY2R, and LeptinR in the hypothalamus. **(A)**, the immunohistochemical slice of AR; **(B)**, the immunohistochemical slice of ERα; **(C)**, the immunohistochemical slice of NPY; **(D)**, the immunohistochemical slice of NPY2R; **(E)**, the immunohistochemical slice of LeptinR. *P< 0.05, ****P< 0.0001.

The expression of AR, ERα, NPY, NPY2R, and leptinR in the ARC of the offspring male rats was observed by quantitative analysis of immunohistochemical images. The results showed that the protein expression levels of ERα, NPY2R, and leptinR in the TG were significantly increased compared with those in the OOG, while the expression levels of AR and NPY in the TG were significantly lower than those in the OOG (*P<* 0.05; **Table 6**). Moreover, the results based on immunohistochemical sections further revealed that the expression of NPY2R and leptinR in the TG was higher than that in the OOG, while the expression of NPY in the TG was lower than that in the OOG. See **Figure 2** for details.

**Table 6:** Comparisons of proteins levels between OOG and TG in the hypothalamus of male offspring ( $\bar{x}$ ± *s*).

Indices	OOG	TG	*F*	*P*
AR	0.273±0.040	0.247±0.032	4.538	<0.05
ERα	0.323±0.030	0.394±0.017	76.953	<0.05
NPY	0.302±0.048	0.273±0.027	4.695	<0.05
NPY2R	0.234±0.027	0.287±0.022	43.167	<0.05
LeptinR	0.179±0.044	0.243±0.037	22.574	<0.05

## 4 Discussion

The early onset of puberty has become a trend. The role of the intrauterine environment during pregnancy in the onset of puberty in offspring has received increasing attention. In the current study, we found that testosterone intervention in pregnant rats leads to an early time of onset of puberty in male offspring rats, a positive association between the time of onset of puberty and AR mRNA, an increase in hypothalamic NPY2R and leptinR protein and a decrease in AR and NPY protein expression. This showed that male offspring rats after androgen intervention during pregnancy might be more sensitive to androgens, leptin, and NPY, thus leading to early puberty.

It has become a consensus that puberty onset is correlated with body shape indicators (37); that is, puberty begins when body weight reaches a certain threshold. In this study, we found positive associations between the time of onset of puberty and weight, body length, and abdominal fat in OOG, which was consistent with previous studies. However, there were positive correlations between the time of onset of puberty and serum DHT, DHEA levels, and FAI in TG, which showed that offspring male rats after androgen intervention during pregnancy might be more sensitive to androgens. At the same time, the protein expression of AR decreased in the TG, which also suggested that the time of onset of puberty of male offspring rats was more sensitive to androgen after testosterone intervention during pregnancy. It follows that the control of puberty onset of male offspring may no longer be the feedback of body shape changes but more through the feedback regulation of sex hormones in offspring male rats after androgen intervention during pregnancy (38).

It is well known that leptin sends a negative feedback signal to the brain center when the body has sufficient energy storage, thereby maintaining the body’s energy balance by reducing food intake and increasing energy expenditure (39). Moreover, related studies have shown that androgen can inhibit the expression of leptin (40). Likewise, in a study on adipocytes of 3T3-L1 mice, it was found that dihydrotestosterone (DHT) significantly decreased leptin transcription and protein expression (41). In normal men, leptin levels may be limited by androgen, resulting in lower leptin levels than in women (42), and if serum leptin levels are above a certain threshold, leptin will in turn inhibit testicular function (43). Taken together, these signs suggest that androgen has an inhibitory effect on leptin. It was found that testosterone intervention reduced the protein expression of AR and upregulated the protein expression of leptinR at the onset of puberty in male offspring rats, which may be caused by the weakening of the inhibitory effect of low levels of androgens on leptin after testosterone intervention during pregnancy. In addition to its role in regulating energy balance, leptin also plays an important role in puberty onset by stimulating gonadotropin expression by upregulating the mRNA levels of GnRHR, activin, and FSHB (44). In this study, the expression of hypothalamic leptinR in the TG was higher than that in the OOG, indicating that the binding of hypothalamic leptin to leptinR may be enhanced after testosterone intervention, which affects the onset time of puberty.

Interestingly, related studies (45) have shown that there is no leptinR protein expression on GnRH neurons; therefore, by which mediator is leptin’s regulation of puberty onset time achieved? NPY, a 36-amino acid orexin-producing protein, is a key feeding center in the hypothalamic arcuate nucleus (46) and binds to its receptors Y1 and Y2 to regulate reproduction and food intake, respectively (47), in which binding to NPY1R can directly inhibit the release of GnRH (28). Moreover, kisspeptin and POMC neurons are also regulated by leptin (48, 49). However, a relevant study showed that increased Kiss1 mRNA levels were detected only after the onset of puberty (50), and Kiss1 does not express substantial amounts of leptin receptors (51), indicating that kisspeptin neurons may play a role after puberty. Another study also noted that the leptin-POMC pathway has sex specificity in terms of reproductive control, that is, it is difficult to play a role in male mice (52). However, for NPY neurons, related studies (29, 53) have provided evidence that serum leptin and hypothalamic leptin receptors are involved in the regulation of GnRH release by NPY and that NPY levels are elevated in leptin-deficient (ob/ob) mice (54). As shown in the results of this study, at the onset of puberty, the protein expression of leptinR and NPY2R in the hypothalamus increased, whereas the expression of NPY protein decreased in the TG, which may be due to the increased sensitivity of offspring male rats to leptin, thus suggesting that the binding of leptin to the leptin receptor was enhanced in the hypothalamus. The signal was transmitted to NPY neurons to inhibit NPY mRNA encoding the NPY protein, resulting in a decrease in NPY protein expression. Furthermore, in the environment of increased expression of NPY2R protein, the binding ability of NPY2R to NPY is enhanced, which mainly plays a role in energy expenditure and locomotion (55); thus, the inhibition of GnRH may be weakened followed by GnRH increasing rapidly during puberty; therefore, puberty starts earlier.

## 5 Limitations

In this study, we detected only NPY2R, not NPY1R, so we cannot draw specific conclusions directly through the binding of NPY and NPY1R. Moreover, the intervention of testosterone was only in the third trimester of pregnancy, and it did not reflect the effect of testosterone on the puberty onset of offspring during pregnancy. At the same time, we sampled at the onset of puberty and reflected the differences between groups in the form of standardization, which may reduce the effectiveness of reflecting differences between groups. Finally, the association results were based on too few samples, which had limitations in the interpretation of associations.

## 6 Conclusion

**Figure 3:**
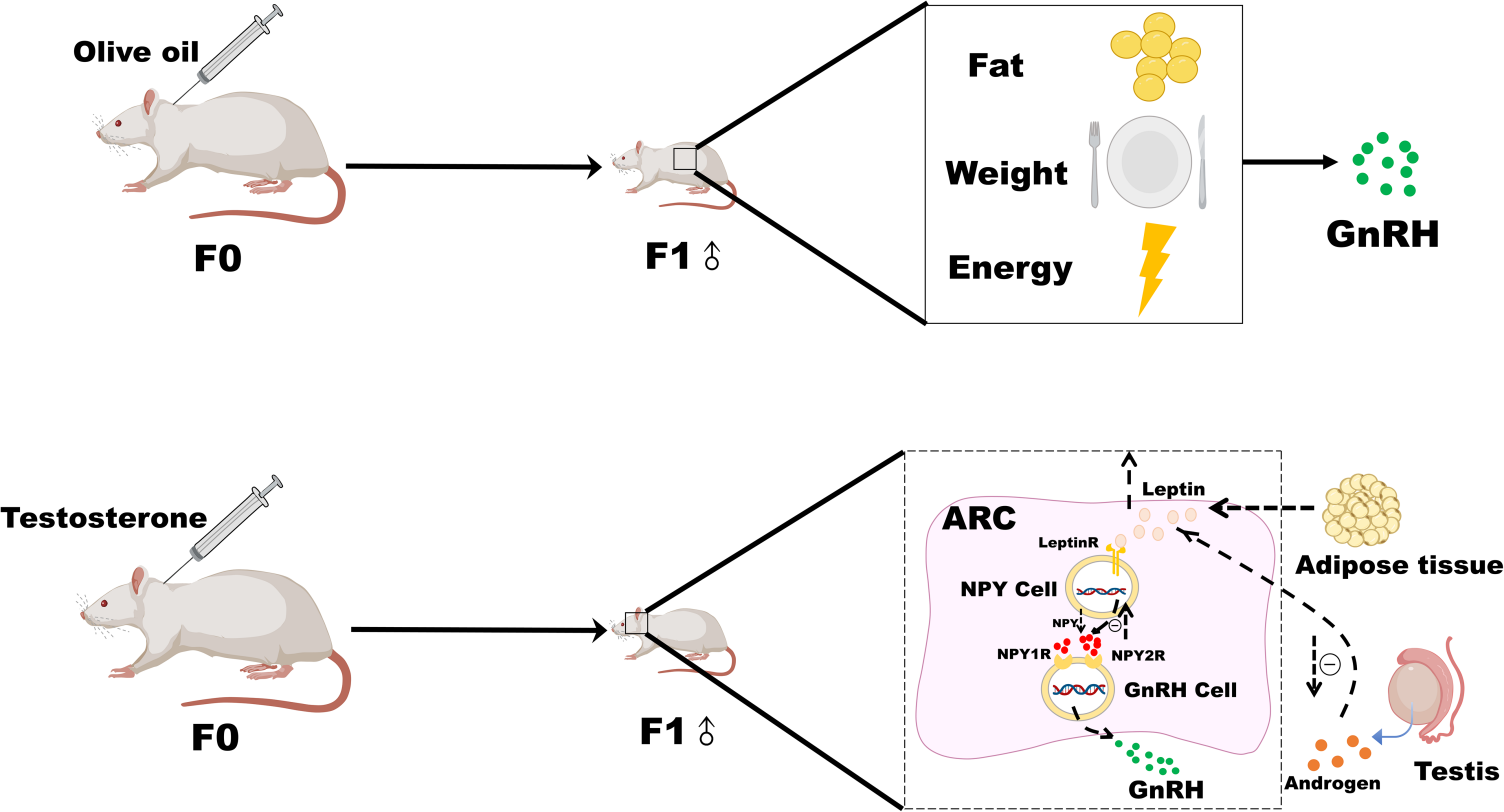
Possible mechanism of leptin-NPY on the onset of puberty in male offspring rats after androgen intervention during pregnancy.

Testosterone intervention in pregnant rats led to an earlier time of onset of puberty in male offspring rats. There were positive correlations between the time of onset of puberty and serum DHT, DHEA levels, and FAI, increased NPY2R mRNA, leptinR, and NPY2R protein expression, and decreased AR and NPY protein expression in male offspring rats after testosterone intervention during pregnancy. These results indicate that high androgen during pregnancy leads to early puberty onset in male offspring, which may be related to the decreased protein expression of AR, the increased expression of leptinR in the hypothalamus, and the enhanced inhibition of NPY neurons, as well as the enhanced binding of NPY2R and NPY. This could render male offspring after testosterone intervention during pregnancy more sensitive to androgens, leptin, and NPY at the onset of puberty. Therefore, the inhibition of GnRH may be weakened, and puberty onset occurs earlier (**Figure 3**).

## Data availability statement

The raw data supporting the conclusions of this article will be made available by the authors, without undue reservation.

## Ethics statement

The animal study was reviewed and approved by Ethics Committee of Bengbu Medical College.

## Author contributions

All authors made substantial contributions to conception and design, acquisition of data, or analysis and interpretation of data; took part in drafting the article or revising it critically for important intellectual content; gave final approval of the version to be published; and agree to be accountable for all aspects of the work.

## References

[1] Livadas S Chrousos GP. Molecular and environmental mechanisms regulating puberty initiation: An integrated approach. Front Endocrinol (Lausanne) (2019) 10:828. doi: 10.3389/fendo.2019.00828 31920956 PMC6915095

[2] Eugster EA. Update on precocious puberty in girls. J Pediatr Adolesc Gynecol (2019) 32(5):455–9. doi: 10.1016/j.jpag.2019.05.011 31158483

[3] Tekgül N Saltık D Vatansever K. Secular trend of menarche age in an immigrant urban city in Turkey: Izmir. Turk J Pediatr (2014) 56(2):138–43. Available at: https://www.turkishjournalpediatrics.org/abstract.php?lang=en&id=1309. 24911846

[4] Song Y Ma J Agardh A Lau PW Hu P Zhang B. Secular trends in age at menarche among Chinese girls from 24 ethnic minorities, 1985 to 2010. Glob Health Action (2015) 8:26929. doi: 10.3402/gha.v8.26929 26220757 PMC4518164

[5] Ong KK Ahmed ML Dunger DB. Lessons from Large population studies on timing and tempo of puberty (Secular trends and relation to body size): The European trend. Mol Cell Endocrinol (2006) 254-255:8–12. doi: 10.1016/j.mce.2006.04.018 16757103

[6] Ohlsson C Bygdell M Celind J Sondén A Tidblad A Sävendahl L. Secular trends in pubertal growth acceleration in Swedish boys born from 1947 to 1996. JAMA Pediatr (2019) 173(9):860–5. doi: 10.1001/jamapediatrics.2019.2315 PMC6647355 31329245

[7] Sørensen K Aksglaede L Petersen JH Juul A. Recent changes in pubertal timing in healthy Danish boys: Associations with body mass index. J Clin Endocrinol Metab (2010) 95(1):263–70. doi: 10.1210/jc.2009-1478 19926714

[8] Ma HM Chen SK Chen RM Zhu C Xiong F Li T. Pubertal development timing in urban Chinese boys. Int J Androl (2011) 34(5 Pt 2):e435–45. doi: 10.1111/j.1365-2605.2011.01173.x 21658069

[9] Barendse MEA Byrne ML Flournoy JC McNeilly EA Guazzelli Williamson V Barrett AY. Multimethod assessment of pubertal timing and associations with internalizing psychopathology in early adolescent girls. J Psychopathol Clin Sci (2022) 131(1):14–25. doi: 10.1037/abn0000721 34941314 PMC9439585

[10] Wang J Kwok MK Au Yeung SL Zhao J Li AM Lam HS. Age of puberty and sleep duration: Observational and mendelian randomization study. Sci Rep (2020) 10(1):3202. doi: 10.1038/s41598-020-59811-9 32081851 PMC7035269

[11] Olgun EG Cetin SK Siklar Z Aycan Z Ozsu E Ceran A. Investigation of early puberty prevalence and time of addition thelarche to pubarche in girls with premature pubarche: Two-year follow-up results. Clin Pediatr Endocrinol (2022) 31(1):25–32. doi: 10.1297/cpe.2021-0042 35002065 PMC8713059

[12] Warmink-Perdijk WDB Peters LL Tigchelaar EF Dekens JAM Jankipersadsing SA Zhernakova A. Lifelines next: A prospective birth cohort adding the next generation to the three-generation lifelines cohort study. Eur J Epidemiol (2020) 35(2):157–68. doi: 10.1007/s10654-020-00614-7 PMC7125065 32100173

[13] Chung DD Pinson MR Bhenderu LS Lai MS Patel RA Miranda RC. Toxic and teratogenic effects of prenatal alcohol exposure on fetal development, adolescence, and adulthood. Int J Mol Sci (2021) 22(16):8785. doi: 10.3390/ijms22168785 34445488 PMC8395909

[14] Aguilera N Salas-Pérez F Ortíz M Álvarez D Echiburú B Maliqueo M. Rodent models in placental research. Implications Fetal Origins Adult Disease Anim Reprod (2022) 19(1):e20210134. doi: 10.1590/1984-3143-ar2021-0134 35493783 PMC9037606

[15] Zhang Q Pei LG Liu M Lv F Chen G Wang H. Reduced testicular steroidogenesis in rat offspring by prenatal nicotine exposure: Epigenetic programming and heritability *Via* Nachr/Hdac4. Food Chem Toxicol (2020) 135:111057. doi: 10.1016/j.fct.2019.111057 31846720

[16] Goldstein JM Hale T Foster SL Tobet SA Handa RJ. Sex differences in major depression and comorbidity of cardiometabolic disorders: Impact of prenatal stress and immune exposures. Neuropsychopharmacology (2019) 44(1):59–70. doi: 10.1038/s41386-018-0146-1 30030541 PMC6235859

[17] Bruni V Capozzi A Lello S. The role of genetics, epigenetics and lifestyle in polycystic ovary syndrome development: The state of the art. Reprod Sci (2022) 29(3):668–79. doi: 10.1007/s43032-021-00515-4 33709373

[18] Gopalakrishnan K Mishra JS Ross JR Abbott DH Kumar S. Hyperandrogenism diminishes maternal-fetal fatty acid transport by increasing Fabp4-mediated placental lipid accumulation†. Biol Reprod (2022) 107(2):514–28. doi: 10.1093/biolre/ioac059 PMC9614932 35357467

[19] Wang Z Yang L Dong H Dong H Cheng L Yi P. Effect of electroacupuncture on the kisspeptin system in a pubertal rat model of polycystic ovary syndrome. Acupunct Med (2021) 39(5):491–500. doi: 10.1177/0964528420971299 33406849

[20] Parsons AM Bouma GJ. A potential role and contribution of androgens in placental development and pregnancy. Life (Basel) (2021) 11(7):644. doi: 10.3390/life11070644 34357016 PMC8305703

[21] Kung KTF Thankamony A Ong KKL Acerini CL Dunger DB Hughes IA. No relationship between prenatal or early postnatal androgen exposure and autistic traits: Evidence using anogenital distance and penile length measurements at birth and 3 months of age. J Child Psychol Psychiatry (2021) 62(7):876–83. doi: 10.1111/jcpp.13335 33049073

[22] Malik IA Durairajanayagam D Singh HJ. Leptin and its actions on reproduction in males. Asian J Androl (2019) 21(3):296–9. doi: 10.4103/aja.aja_98_18 PMC6498734 30539926

[23] Ohga H Matsuyama M. *In vitro* action of leptin on gonadotropin secretion in pre-pubertal Male chub mackerel. Comp Biochem Physiol A Mol Integr Physiol (2021) 253:110856. doi: 10.1016/j.cbpa.2020.110856 33249145

[24] Chen X Xiao Z Cai Y Huang L Chen C. Hypothalamic mechanisms of obesity-associated disturbance of hypothalamic-Pituitary-Ovarian axis. Trends Endocrinol Metab (2022) 33(3):206–17. doi: 10.1016/j.tem.2021.12.004 35063326

[25] Nieuwenhuis D Pujol-Gualdo N Arnoldussen IAC Kiliaan AJ. Adipokines: A gear shift in puberty. Obes Rev (2020) 21(6):e13005. doi: 10.1111/obr.13005 32003144 PMC7317558

[26] Odle AK Akhter N Syed MM Allensworth-James ML Beneš H Melgar Castillo AI. Leptin regulation of gonadotrope gonadotropin-releasing hormone receptors as a metabolic checkpoint and gateway to reproductive competence. Front Endocrinol (Lausanne) (2017) 8:367. doi: 10.3389/fendo.2017.00367 29354094 PMC5760501

[27] Coutinho EA Prescott M Hessler S Marshall CJ Herbison AE Campbell RE. Activation of a classic hunger circuit slows luteinizing hormone pulsatility. Neuroendocrinology (2020) 110(7-8):671–87. doi: 10.1159/000504225 31630145

[28] Hessler S Liu X Herbison AE. Direct inhibition of arcuate kisspeptin neurones by neuropeptide y in the Male and female mouse. J Neuroendocrinol (2020) 32(5):e12849. doi: 10.1111/jne.12849 32337804

[29] Guzmán A Hernández-Coronado CG Rosales-Torres AM Hernández-Medrano JH. Leptin regulates neuropeptides associated with food intake and gnrh secretion. Ann Endocrinol (Paris) (2019) 80(1):38–46. doi: 10.1016/j.ando.2018.07.012 30243474

[30] Naulé L Maione L Kaiser UB. Puberty, a sensitive window of hypothalamic development and plasticity. Endocrinology (2021) 162(1):bqaa209. doi: 10.1210/endocr/bqaa209 33175140 PMC7733306

[31] Kaya A Orbak Z Polat İ Polat H Gümüşdere M. Leptin and neuropeptide y levels in newborns. J Pediatr Endocrinol Metab (2016) 29(1):21–5. doi: 10.1515/jpem-2015-0201 26353170

[32] Marraudino M Bo E Carlini E Farinetti A Ponti G Zanella I. Hypothalamic expression of neuropeptide y (Npy) and pro-opiomelanocortin (Pomc) in adult Male mice is affected by chronic exposure to endocrine disruptors. Metabolites (2021) 11(6):368. doi: 10.3390/metabo11060368 34207679 PMC8228876

[33] Marshall CJ Prescott M Campbell RE. Investigating the Npy/Agrp/Gaba to gnrh neuron circuit in prenatally androgenized pcos-like mice. J Endocr Soc (2020) 4(11):bvaa129. doi: 10.1210/jendso/bvaa129 33094210 PMC7566551

[34] Bano R Shamas S Khan SUH Shahab M. Inverse age-related changes between hypothalamic npy and Kiss1 gene expression during pubertal initiation in Male rhesus monkey. Reprod Biol (2022) 22(1):100599. doi: 10.1016/j.repbio.2021.100599 35033902

[35] Guzelkas I Orbak Z Doneray H Ozturk N Sagsoz N. Serum kisspeptin, leptin, neuropeptide y, and neurokinin b levels in adolescents with polycystic ovary syndrome. J Pediatr Endocrinol Metab (2022) 35(4):481–7. doi: 10.1515/jpem-2021-0487 35170267

[36] True C Takahashi D Kirigiti M Lindsley SR Moctezuma C Arik A. Arcuate nucleus neuropeptide coexpression and connections to gonadotrophin-releasing hormone neurones in the female rhesus macaque. J Neuroendocrinol (2017) 29(6):10.1111/jne.12491. doi: 10.1111/jne.12491 PMC5523807 28561903

[37] Dai X Pu D Wang L Cheng X Liu X Yin Z. Emergence of breeding tubercles and puberty onset in Male zebrafish: Evidence for a dependence on body growth. J Fish Biol (2021) 99(3):1071–8. doi: 10.1111/jfb.14811 34037242

[38] Morford J Mauvais-Jarvis F. Sex differences in the effects of androgens acting in the central nervous system on metabolism. Dialogues Clin Neurosci (2016) 18(4):415–24. doi: 10.31887/DCNS.2016.18.4/fmauvais PMC5286727 28179813

[39] Maffei M Giordano A. Leptin, the brain and energy homeostasis: From an apparently simple to a highly complex neuronal system. Rev Endocr Metab Disord (2022) 23(1):87–101. doi: 10.1007/s11154-021-09636-2 33822303

[40] Khodamoradi K Parmar M Khosravizadeh Z Kuchakulla M Manoharan M Arora H. The role of leptin and obesity on Male infertility. Curr Opin Urol (2020) 30(3):334–9. doi: 10.1097/mou.0000000000000762 32205811

[41] Jenks MZ Fairfield HE Johnson EC Morrison RF Muday GK. Sex steroid hormones regulate leptin transcript accumulation and protein secretion in 3t3-L1 cells. Sci Rep (2017) 7(1):8232. doi: 10.1038/s41598-017-07473-5 28811502 PMC5558017

[42] Liu Y Xu YC Cui YG Jiang SW Diao FY Liu JY. Androgen excess increases food intake in a rat polycystic ovary syndrome model by downregulating hypothalamus insulin and leptin signaling pathways preceding weight gain. Neuroendocrinology (2022) 112(10):966–81. doi: 10.1159/000521236 PMC9677863 34847556

[43] Childs GV Odle AK MacNicol MC MacNicol AM. The importance of leptin to reproduction. Endocrinology (2021) 162(2):bqaa204. doi: 10.1210/endocr/bqaa204 33165520 PMC7749705

[44] D'Occhio MJ Baruselli PS Campanile G. Influence of nutrition, body condition, and metabolic status on reproduction in female beef cattle: A review. Theriogenology (2019) 125:277–84. doi: 10.1016/j.theriogenology.2018.11.010 30497026

[45] Ohga H Ito K Kakino K Mon H Kusakabe T Lee JM. Leptin is an important endocrine player that directly activates gonadotropic cells in teleost fish, chub mackerel. Cells (2021) 10(12):3505. doi: 10.3390/cells10123505 34944013 PMC8700583

[46] Vohra MS Benchoula K Serpell CJ Hwa WE. Agrp/Npy and pomc neurons in the arcuate nucleus and their potential role in treatment of obesity. Eur J Pharmacol (2022) 915:174611. doi: 10.1016/j.ejphar.2021.174611 34798121

[47] Yuan D Gao Y Zhang X Wang B Chen H Wu Y. Npy and npy receptors in the central control of feeding and interactions with cart and Mc4r in Siberian sturgeon. Gen Comp Endocrinol (2019) 284:113239. doi: 10.1016/j.ygcen.2019.113239 31394086

[48] Bohlen TM de Paula DG Teixeira PDS da Silva Mansano N Andrade Alves G Donato J Jr.. Socs3 ablation in kisspeptin cells partially prevents lipopolysaccharide-induced body weight loss. Cytokine (2022) 158:155999. doi: 10.1016/j.cyto.2022.155999 35985175

[49] Turkkahraman D Sirazi EC Aykal G. Serum alpha-Melanocyte-Stimulating hormone (a-msh), brain-derived neurotrophic factor (Bdnf), and agouti-related protein (Agrp) levels in children with prader-willi or bardet-biedl syndromes. J Endocrinol Invest (2022) 45(5):1031–7. doi: 10.1007/s40618-021-01737-8 35098494

[50] Yan X Yuan C Zhao N Cui Y Liu J. Prenatal androgen excess enhances stimulation of the gnrh pulse in pubertal female rats. J Endocrinol (2014) 222(1):73–85. doi: 10.1530/joe-14-0021 24829217

[51] Donato J Jr. Cravo RM Frazão R Gautron L Scott MM Lachey J. Leptin's effect on puberty in mice is relayed by the ventral premammillary nucleus and does not require signaling in Kiss1 neurons. J Clin Invest (2011) 121(1):355–68. doi: 10.1172/jci45106 PMC3007164 21183787

[52] Manfredi-Lozano M Roa J Ruiz-Pino F Piet R Garcia-Galiano D Pineda R. Defining a novel leptin-Melanocortin-Kisspeptin pathway involved in the metabolic control of puberty. Mol Metab (2016) 5(10):844–57. doi: 10.1016/j.molmet.2016.08.003 PMC5034608 27688998

[53] Chu SC Chen PN Chen JR Yu CH Hsieh YS Kuo DY. Role of hypothalamic leptin-leprb signaling in npy-Cart-Mediated appetite suppression in amphetamine-treated rats. Horm Behav (2018) 98:173–82. doi: 10.1016/j.yhbeh.2017.12.019 29307696

[54] Padilla SL Qiu J Nestor CC Zhang C Smith AW Whiddon BB. Agrp to Kiss1 neuron signaling links nutritional state and fertility. Proc Natl Acad Sci U.S.A. (2017) 114(9):2413–8. doi: 10.1073/pnas.1621065114 PMC5338482 28196880

[55] Qi Y Lee NJ Ip CK Enriquez R Tasan R Zhang L. Npy derived from agrp neurons controls feeding *via* Y1 and energy expenditure and food foraging behaviour *via* Y2 signalling. Mol Metab (2022) 59:101455. doi: 10.1016/j.molmet.2022.101455 35167990 PMC8886056

